# Supplementary material for: Capsaicin Modulates Hepatic and Intestinal Inflammation and Oxidative Stress by Regulating the Colon Microbiota
Source: Antioxidants (Basel). 2024 Aug 2;13(8):942. doi: 10.3390/antiox13080942 (PMC11352159; doi:10.3390/antiox13080942)
Supplement: Supplementary file 1 [file antioxidants-13-00942-s001.zip › antioxidants-3080967-supplementary.pdf]

Supplementary Figure S1

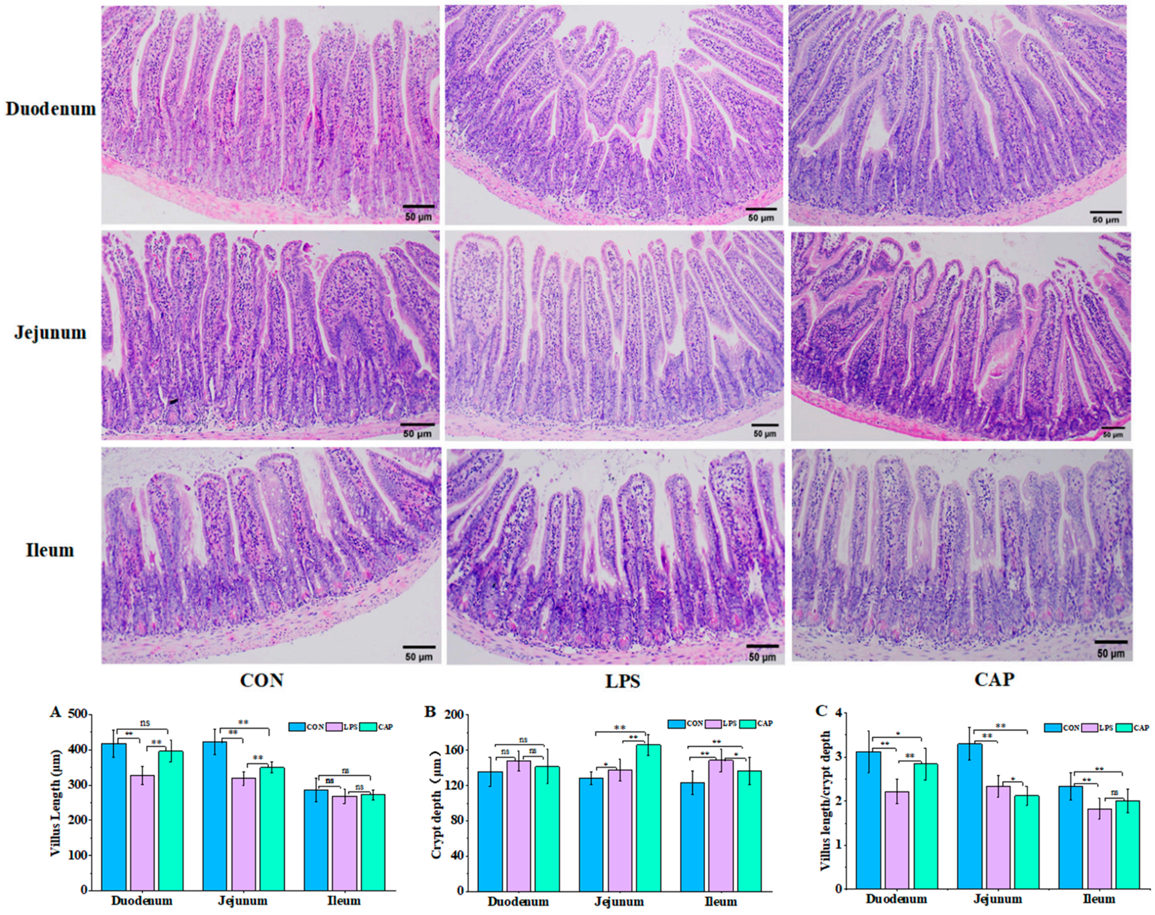

Figure S1. Effect of CAP on the morphology of mouse intestinal tissues. The effect of CAP on the intestinal was assessed by H&E staining. Original magnification: ×100; (A) Length of the duodenum, jejunum, and ileum villi. (B) Depth of the duodenal, jejunal and ileal crypts. (C) Ratio of duodenal, jejunal, and ileal villus length to crypt depth.

Note: ns indicates that the difference was not significant (p>0.05), * p< 0.05, ** p< 0.01.

Supplementary Figure S2

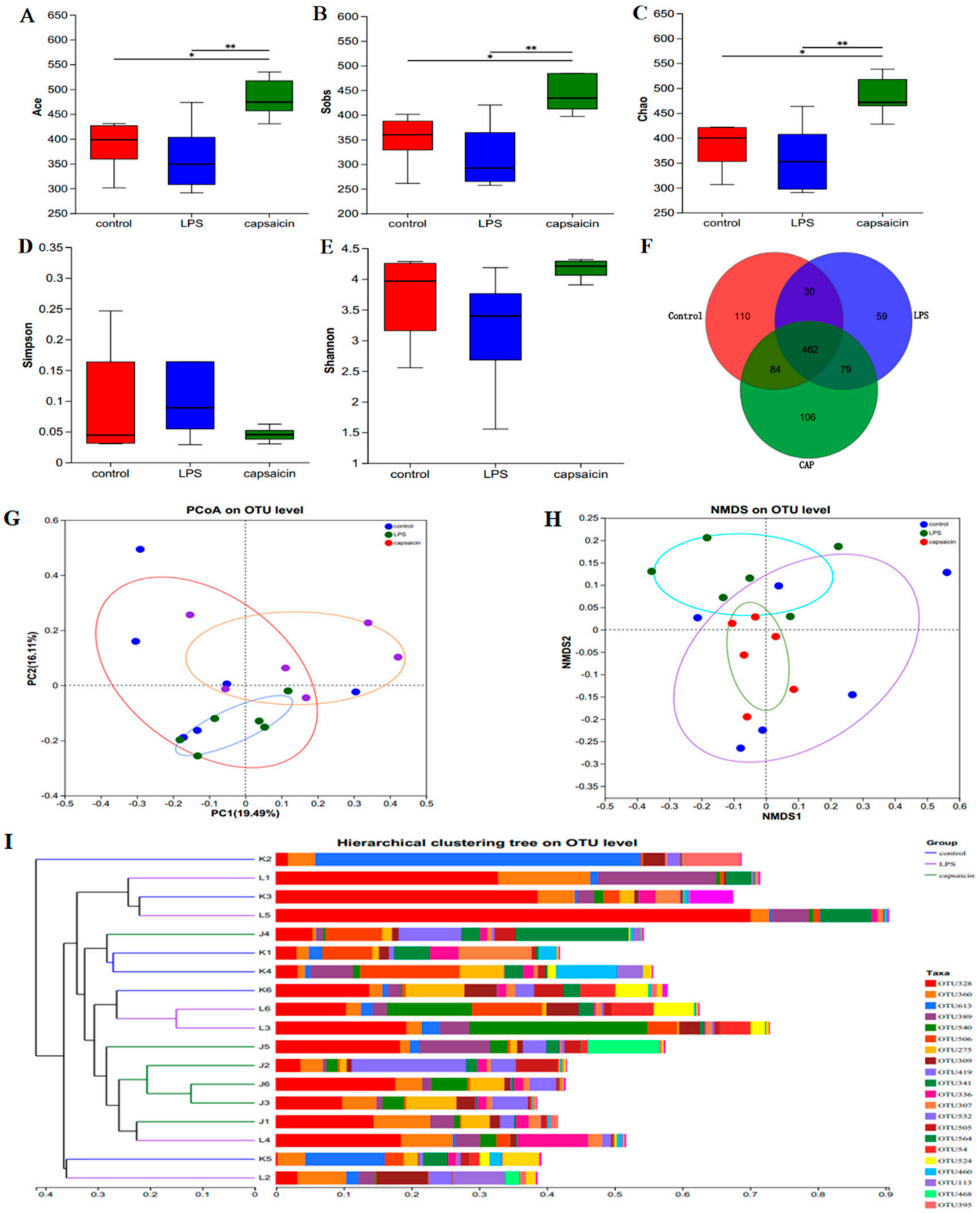

Figure S2. Effect of CAP on the microbial composition of mouse colon. (A) Ace, (B) Sobs, (C) Chao, (D) Simpson, and (E) Shannon indices. (F) Venn diagram showing the species similarity. (G) PCoA. (H) NMDS. (I) Hierarchical cluster analysis.

Note: ns indicates that the difference was not significant (p>0.05), * p<0.05, ** p<0.01.
